# Characterization of a liposome electrokinetic capillary chromatography system for surrogation of cell membrane partitions

**DOI:** 10.1007/s00216-026-06365-w

**Published:** 2026-03-03

**Authors:** Paula Vidal, Elisabet Fuguet, Martí Rosés

**Affiliations:** 1 Department of Analytical Chemistry and Institute of Biomedicine of the University of Barcelona (IBUB), Universitat de Barcelona, Martí i Franquès 1-11, 08028 Barcelona, Spain; 2 Serra Húnter Fellow, Generalitat de Catalunya, Barcelona, Spain

**Keywords:** Liposome electrokinetic capillary chromatography, Abraham solvation parameter model, Biomimetic chromatography, Linear solvation energy relationships, Biological surrogation

## Abstract

The solute-solvent interactions that determine distribution between water and a model liposome, representative of biological cell membranes, are studied by liposome electrokinetic chromatography (LEKC) and characterized and quantified by the Abraham Solvation Parameter Model. Because of the limited solubility of usual liposome markers, several methods to determine the liposome mobility, required to calculate solute mass distribution ratios, have been tested and a new one, based on the Abraham model applied to homologous series, is proposed with successful results. Characterization of the LEKC system by the Abraham model shows that the most significant interactions ruling liposome partition are the creation of solute cavities in the phases, which is easier in the liposome than in water, and hydrogen bonding from liposome and water phases to the solute (solute hydrogen bond basicity), which is stronger from the aqueous phase. To a minor degree, solute dipolarity and hydrogen bond acidity (hydrogen bonding from solutes to phases) contribute to partition too. Dipolarity interactions favor partition to water rather than to the liposome. However, and contrary to solute hydrogen bond basicity, solute hydrogen bond acidity favors partition to the liposome, revealing that the studied liposome is a better hydrogen bond acceptor than water. Polarizability interactions by solute *n* and *π* electrons are not significant. Comparison of the LEKC system Abraham characterization parameters to those of several in vivo and in vitro blood-organ partitions, characterized by the same model, indicates that the studied liposome system can be a good surrogate of blood-skin partition.

**Graphical abstract:**

**Figure:**
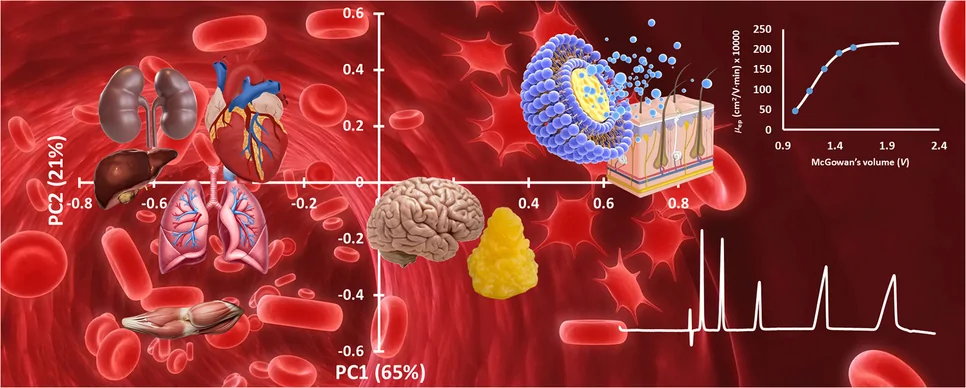

**Supplementary Information:**

The online version contains supplementary material available at https://doi.org/10.1007/s00216-026-06365-w.

## Introduction

Biomimetic chromatography is an emerging mode of liquid chromatography, usually with proteins or phospholipids immobilized in a silica support. It mimics biological environments and hence models the in vivo transport, cell permeation, distribution of drugs, and other biopartitions. Biomimetic chromatography minimizes the need for animal experiments, with their ethical concerns, reduces costs in terms of time, money, and resources [1–5] and thus improves the selection of the most promising candidates in the drug discovery process.

Liposome electrokinetic capillary chromatography (LEKC) is a variant of biomimetic chromatography, based on capillary electrophoresis technique, that uses liposomes as pseudostationary phases. Because phospholipids are a major constituent of cell membranes, LEKC is an excellent technique to study drug interactions with biomimetic membranes. Indeed, an adequate modeling of the composition of the carrier liposome may mimic a specific biological environment and thus surrogate cell permeation or other biological distribution processes [6–10]. Since a variety of natural and synthetic phospholipids can be used to create liposomes, the combination of this feasibility with the intrinsic advantages of capillary electrophoresis (no need of high amounts of drug sample, no need of high sample purity, variety of detection techniques, low consumption of chemicals and solvents, high separation efficiency, and short analysis time [11]) makes LEKC a versatile and powerful tool in the early stages of drug discovery and development.

In biopartitions, the bioactive compound distributes between biological aqueous and organic phases (e.g., blood/brain distribution). Usually, the two phases of the biomimetic system are prepared to mimic the composition and properties of the biological system to emulate. For instance, in biomimetic LEKC, the liposome composition of the pseudostationary phase is chosen to match as much as possible that of the organic phase (e.g., brain cells) and the mobile phase to match the biological aqueous or polar phase (e.g., water buffered at the physiological pH of 7.4 for blood emulation). However, the composition of biological phases is very complex and difficult to emulate. Then, the goodness of the surrogation is evaluated by empiric correlations between the biological property of interest (e.g., blood/brain distribution constant) and the measured biomimetic parameter (e.g., chromatographic mass distribution ratio), which often are fair at most [10] and many times require the inclusion of additional descriptors.

Whenever possible, a better approach is to characterize the solute-solvent interactions that rule the biological process and model the biomimetic system to have similar ones. This procedure requires the biological and biomimetic systems to be characterized by the same model and compare their similarity [12]. An additional advantage of this procedure is that it does not need the biological and biomimetic data for the same compounds, as in the empirical correlations because these data are not directly compared, only the system constants of the models. Moreover, when the system constants of several biological and biomimetic systems are known, the best surrogation biomimetic system for a particular biological process can be selected, or the usefulness of a specific biomimetic system to surrogate different biological processes evaluated.

In this paper, we evaluate the feasibility of a model liposome to surrogate several blood/organ biopartition processes by LEKC. The liposome (PC_49_/PA_43_/C_8_) is constituted by phosphatidylcholine 49% mol/mol (PC_49_), palmitic acid 43% mol/mol (PA_43_), and cholesterol 8% mol/mol (C_8_). Phosphatidylcholine is the most abundant phospholipid in human tissues [13, 14]. It is a zwitterionic compound, but uncharged at the physiological pH of 7.4, which prevents its direct use in LEKC. Thus, it has been combined with palmitic acid, a fatty acid very common in lipid components and the most abundant in our bodies [15, 16], which is negatively charged at pH 7.4. In addition to phospholipids and fatty acids, sterols are also important components of cell membranes. In mammals, the predominant sterol is cholesterol. Although sterols cannot form bilayers by themselves, they can be inserted into the bilayers of liposomes, with the hydroxyl group oriented to the aqueous media and the aliphatic chain aligned with the lipidic hydrocarbon chains. Thus, they can form more fluid bilayers and stabilize the cell membrane [16].

The solute-solvent interactions in the PC_49_/PA_43_/C_8_ LEKC system are characterized by the model of Abraham, or Solvent Parameter Model, a well-known Linear Free Energy Relationships (LFER) model widely used to characterize many physicochemical and biological systems. Characterization of the LEKC Abraham system constants allows comparison to the bibliographic biological system constants and evaluation of the feasibility of the surrogation [12].

Analysis by LFER models requires the measurement of a property directly related to the free energy change of the process, which usually is the constant of the process (in logarithmic form). However, chromatographic constants are difficult to measure because the phase ratio must be known to calculate them from the measured retention parameters. Instead, the mass distribution ratio (*k*_LEKC_) [17] is used in electrokinetic chromatography because it is directly related to the measured electrophoretic mobilities of analyte $\left(\mu_{ep}\right)$ and liposome $\left(\mu_{lip}\right)$ through Eq. (1).

1 $${k}_{\mathrm{LEKC}}=\frac{{\mu }_{\mathrm{ep}}}{{\mu }_{\mathrm{lip}}-{\mu }_{\mathrm{ep}}}$$

Correct determination of the right migration time of liposomes (directly related to its mobility) is difficult because of the limited solubility of usual EKC markers. Several methods have been tested and a new one, based on the LFER of Abraham, is developed.

## Theory

### The LFER model of Abraham

The LFER models of Abraham are QSAR (Quantitative Structure Activity Relationships) or QSPR (Quantitative Structure Property Relationships) that relate by a multilinear equation a biological or physicochemical property of a compound (directly related to its free energy variation in the process) to a series of descriptors that depend on solute’s structure. Abraham equations are in general applied to solute-solvent interactions. Each term of the multilinear equation reflects a particular solute-solvent interaction [18] denoted by the product of a solute and a solvent descriptor. Solute descriptors are written in capital letters and the corresponding solvent parameters, which are called system constants [19], by lowercase letters. The main general Abraham equation, developed for neutral solutes, considers five kinds of interactions: creation of a cavity in the solvent to accommodate the solute (*v*V); hydrogen bond donation from solvent to solute (*b*B); hydrogen bond donation from solute to solvent (*a*A); general interactions between dipoles and induced dipoles (*s*S); and polarizability contributions from solute *n* and *π* electrons (*e*E), and takes the form of Eq. (2).

2 $$\mathrm{log}SP=c+e\mathrm{E}+s\mathrm{S}+a\mathrm{A}+bB+v\mathrm{V}$$

In Eq. (2), *SP* represents the measured biological or physicochemical parameter directly related to the free energy change, such as the distribution constant between water and liposomes, or between blood and organs. In LEKC, as in many liquid chromatography techniques, distribution constants are very difficult to determine because the volume of the stationary phase (in HPLC) or pseudostationary phase (in EKC) cannot be directly measured. Consequently, retention factors (in HPLC) or mass distribution ratios (e.g., *k*_LEKC_) are used instead, as they are directly related to the distribution constant through the phase ratio, which is assumed to be constant. The constant term (*c*) accounts for system factors that are not dependent on the solute-solvent interactions (e.g., normalization of solute descriptors, property unit changes, or conversion factors, such as phase ratio in HPLC or EKC). E, S, A, B, and V are the solute molecular descriptors of excess molar refraction (in reference to a linear alkane of same molecular size), dipolarity/polarizability, hydrogen bond acidity, hydrogen bond basicity, and McGowan’s characteristic volume (in cm^3^ mol^−1^/100), respectively. These solute descriptors have been established for more than 4000 solutes and reported in free [20] and payment [21] databases. Thus, to establish an Abraham model for a particular system, the SP property is measured for a set of solutes of known descriptors, and the system constants *e*, *s*,* a*, *b*, and *v* are obtained by multiple linear regression of SP against the solute descriptors. In partition processes, as LEKC and many biological systems, the system constants reflect the differences of solute interaction between the two phases (e.g., running buffer-liposome, blood-brain). More detailed information on the procedure and requirements of solute descriptors for a good correlation analysis are given elsewhere [19].

The Abraham model has been applied to a host of physicochemical and biological systems [20–23] and there is a corpus of bibliographic data on system constants, which allows comparison of these systems. In particular, new parametrized systems, like the one of this study, can be compared to all these data to establish its similarity and the feasibility of biological surrogation.

### Comparison of LFER models

For a good surrogation of a biological system by a chromatographic one, a linear relationship between the biological and chromatographic measured parameters is expected. This fact implies the system constants of the biological and chromatographic Abraham models to be proportional [12], i.e., the normalized system constants must be very similar. Normalized system constants are obtained by dividing each system constant by the module, or length, of the correlation vector. In this instance, the Abraham equation can be written as in Eq. (3):

3 $$\mathrm{log}SP=c+l({e}_{u}\mathrm{E}+{s}_{u}\mathrm{S}+{a}_{u}\mathrm{A}+{b}_{u}\mathrm{B}+{v}_{u}\mathrm{V})$$

where the subscript u denotes the components of the unitary correlation vector or normalized system constants, and* l* is the length of the correlation vector defined in Eq. (4).

4 $$l=\sqrt{{e}^{2}+{s}^{2}+{a}^{2}+{b}^{2}+{v}^{2}}$$

The similarity between the normalized system constants of the biological (subscript SP) and LEKC (subscript LEKC) Abraham correlations can be evaluated by measuring the distance *d* between them according to Eq. (5).

5 $$d=\sqrt{{\left({e}_{uSP}{-e}_{uLEKC}\right)}^{2}+{\left({s}_{uSP}{-s}_{uLEKC}\right)}^{2}+{\left({a}_{uSP}{-a}_{uLEKC}\right)}^{2}{+\left({b}_{uSP}{-b}_{uLEKC}\right)}^{2}+{\left({v}_{uSP}{-v}_{uLEKC}\right)}^{2}}$$

A distance close to zero would indicate that a very good correlation between the biological SP parameter and the measured LEKC parameter is expected. A *d* limit of 0.25 has been proposed for good or acceptable expected surrogations [12]. Principal component analysis (PCA) and dendrograms using *d* as a distance metric can be very helpful to visualize the similarity of the compared systems and cluster the similar ones (i.e., with *d* < 0.25) [12].

## Materials and methods

### Instrumentation

Electrokinetic measurements were performed with a CE7100 capillary electrophoresis system (Agilent Technologies, USA). Liposome synthesis involved an R-200 rotary evaporator (Büchi, Switzerland), an ultrasonic bath (J.P Selecta, Spain), and an extruder (Northern Lipids INC., Canada) connected to a Termotronic-100 water bath (J.P. Selecta, Spain). The size of the synthesized vesicles was determined using a Zetasizer Nano ZSP (Malvern Panalytical, UK/Netherlands). The pH of the buffer solutions was measured with a GLP 22 pH meter (Crison, Spain) equipped with an EL170 electrode (Accsen Instrumental, Spain).

### Reagents

Liposome preparation employed phosphatidylcholine (95%, Avanti Polar Lipids, USA), cholesterol (> 99%, Sigma, USA), and palmitic acid (> 99%, Merck, Germany). For the Abraham characterization of the PC_49_/PA_43_/C_8_ system, solutes (purity > 98%) from various suppliers (Baker, USA; Merck, Germany; Sigma-Aldrich, USA; TCI, Japan; Carlo Erba, Italy; Alfa Aesar, USA; Fluka, Switzerland; Scharlau, Spain; and Riedel-de Haën, Germany) were used. The complete list of these solutes is provided in Table SI1 of the Supplementary Information. Methanol (HPLC grade, ITW Reagents, Spain), chloroform (> 99%, Fisher, USA), sodium monohydrogen phosphate (> 99%, Baker, USA), and hydrochloric acid (37%, VWR, USA) were also employed.

### Sample preparation

Solute solutions were prepared at concentrations of 1000 or 2000 mg L^−1^ in a mixture of 20 mM Na_2_HPO_4_ buffer (pH 7.4) and methanol. The proportion of methanol, adjusted according to the solubility of each solute, was maintained at or below 60% (v/v).

### Procedure

#### Liposome preparation

Model liposomes were formulated with a molar composition of 49% phosphatidylcholine, 8% cholesterol, and 43% palmitic acid at a total lipid concentration of 15 mM, with palmitic acid conferring a net negative surface charge. Lipids were dissolved in 10 mL of a chloroform:methanol mixture (9:1, v/v). The solvents were then removed under reduced pressure at 70 °C using a rotary evaporator for 20 min, yielding a thin, homogeneous lipid film on the flask walls.

The lipid film was rehydrated with 20 mL of 20 mM Na_2_HPO_4_ buffer (pH 7.4, adjusted with 1 M HCl) and agitated for 5 min on the rotary evaporator without vacuum to facilitate lipid dispersion. The resulting suspension was ultrasonicated to generate multilamellar vesicles.

To obtain homogeneous unilamellar liposomes, the suspension was extruded at 60 °C, above the lipid phase transition temperature, through polycarbonate membranes in three consecutive steps: five passes through 200 nm filters, ten passes through 100 nm filters, and ten passes through 50 nm filters. Liposomes were freshly prepared every 48 h and stored at room temperature until use. Their hydrodynamic diameter was determined by dynamic light scattering, yielding a mean value of 74 ± 5 nm and a polydispersity index of 0.12 ± 0.05. A stability study showed that liposome size increased over time, which affected electrophoretic mobility. Consequently, a single liposome mobility value was not assumed. Instead, it was determined daily. Further details on the stability study and the corresponding data are provided in the Supplementary Information.

#### Electrokinetic measurements

Electrokinetic measurements were performed using a fused silica capillary (Polymicro Technologies, USA) with an inner diameter of 50 µm, an outer diameter of 375 µm, a total length of 48.5 cm, and an effective length of 40 cm. Prior to first use, the capillary was activated with a 0.5 M NaOH solution for 10 min to generate silanol groups on the inner surface.

For daily preconditioning, the capillary was sequentially rinsed with methanol (5 min), ultrapure water (5 min), 0.1 M NaOH (5 min), and ultrapure water (2 min). A liposome suspension or, depending on the experimental protocol, a solution of 20 mM Na_2_HPO_4_ buffer (pH 7.4) was then run for 15 min.

During analysis, the capillary cassette was maintained at 37 °C. Samples were injected hydrodynamically at 50 mbar for 5 s. Separations were performed at an applied voltage of 20 kV, under which no Joule heating was observed. Methanol was used as the electroosmotic flow marker. Before each injection, the capillary was sequentially rinsed with ultrapure water (1 min), 0.1 M NaOH (1 min), and ultrapure water (1 min), followed by either liposome suspension or 20 mM Na_2_HPO_4_ buffer (2 min). Detection was performed using a UV-visible detector at 210, 214, and 280 nm.

## Calculations

All calculations were done in Microsoft Excel (Microsoft Corporation, USA). Electrophoretic mobilities of solutes $\left(\mu_{ep}\right)$ and liposomes $\left(\mu_{lip}\right)$ were calculated according to Eq. (6) from the electroosmotic hold-up time (*t*_0_), the migration time of the solutes or the liposome marker (*t*_m_), the applied voltage (*U*), and the total (*L*) and effective (*l*) capillary lengths.

6 $$\mu =\frac{lL}{U}\left(\frac{1}{{t}_{\mathrm{m}}}-\frac{1}{{t}_{0}}\right)$$

Non-linear regressions were performed by the Solver tool of Excel and the statistics of the fittings were calculated through the Excel macro “Ref_GN_LM”, which is based on the Levenberg-Marquardt modification of the Gauss-Newton non-linear least-squares iterative algorithm [24].

Past 4.03 program was used for dendrogram and principal component analysis. Clustering was done by the classical multivariate method using Paired Group (UPGMA) algorithm and Euclidean similarity index [25]. The Abraham experimental descriptor values were obtained from Percepta software (ACDLabs, Toronto, Canada) [21].

## Results and discussion

### Determination of liposome mobilities

Several methods were explored to determine liposome migration time and mobility using dodecanophenone as the liposome marker. Preliminary solubility tests showed that dodecanophenone does not dissolve enough to obtain measurable peaks in a mixture of Na_2_HPO_4_ buffer and 60% (v/v) methanol, the highest methanol proportion that does not disrupt liposomes. To overcome this limitation, four alternative approaches were tested: two based on capillary zone electrophoresis (CZE) and two on liposome electrokinetic chromatography (LEKC).

**Fig. 1:**
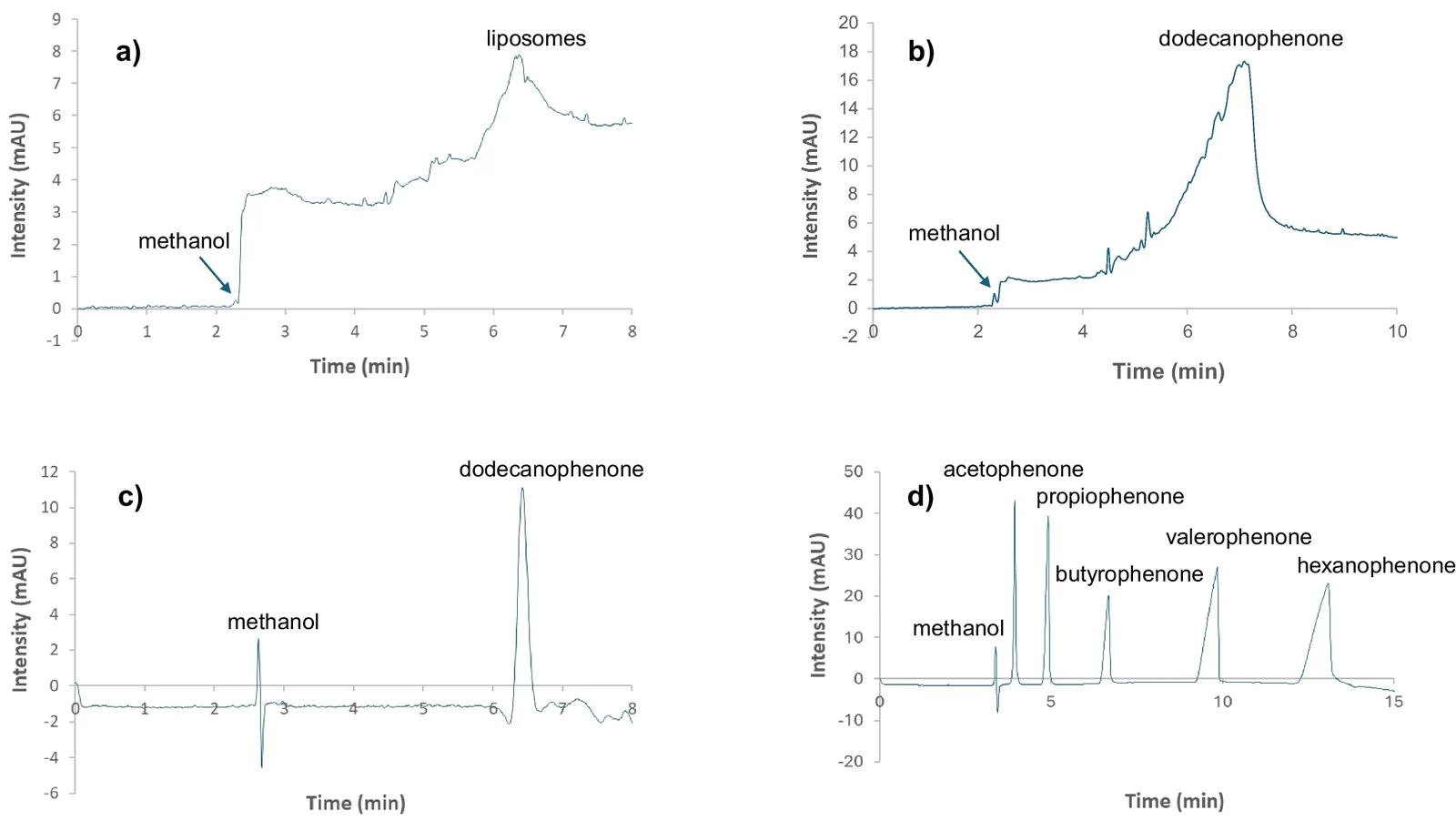
Electropherograms obtained in the determination of the liposome migration time according to different methods. **a** Injection of liposomes solution with 5% methanol and running in CZE mode (only buffer at pH 7.4). **b** Injection of dodecanophenone solved in liposomes suspension and 5% methanol and running in CZE mode. **c** Injection of dodecanophenone solved in liposomes and 5% methanol and running in LEKC mode. **d** Injection of a mixture of alkylphenones solved in 60% methanol and running in LEKC mode

In the first method, liposomes were directly injected adding 5% methanol (EOF marker) to the liposome solution, using 20 mM Na_2_HPO_4_ buffer at pH 7.4 as the electrolyte [26] in CZE mode. In the second and third methods, a 2000 mg L^−1^ solution of dodecanophenone solved in liposome suspension containing 5% methanol was injected. The second method used 20 mM Na_2_HPO_4_ buffer (pH 7.4) as the electrolyte (CZE mode), whereas the third employed liposome suspension (LEKC mode). Electropherograms obtained using the first two methods, shown in Fig. 1a and b, exhibited poorly resolved signals, with the methanol peak difficult to distinguish and the liposome or dodecanophenone peak appearing broad and irregular. In contrast, the third method, illustrated in Fig. 1c, produced sharp, well-defined peaks for methanol and dodecanophenone, which leads to clearer and more interpretable results. However, despite this improvement, all three methods showed limited reproducibility.

The three methods also revealed differences in the calculated mobility of the liposomes, ${\mu }_{\mathrm{lip}}$ in (cm^2^/(V min)) × 10,000. Results from the first two methods, both based on CZE, were in close agreement (274.4 ± 0.2; 279 ± 3) but differed from those obtained with the third method, using LEKC (218 ± 2). This discrepancy can be attributed to the fact that electrophoretic mobility depends on the viscosity of the supporting electrolyte, i.e., the addition of liposome in LEKC mode changes viscosity of the electrolyte [27]. As the system characterization was performed using LEKC, the third method was considered the most representative for this study.

Although the third method provided the most representative measurements, all three approaches exhibited important limitations. The first two methods showed poor peak definition and low reproducibility, whereas the third method, despite providing sharper peaks, still showed reduced reproducibility and was further limited by the low aqueous solubility of dodecanophenone. These combined limitations motivated the proposal of a fourth method, specifically designed to overcome both reproducibility and solubility constraints.

This new approach, also based on LEKC, follows a previous general method proposed for determination of hold-up times in liquid chromatography [28, 29]. In this approach, liposome mobility $({\mu }_{\mathrm{lip}})$ was determined by extrapolating the electrophoretic mobility $({\mu }_{\mathrm{ep}})$ of a homologous series of compounds with increasing hydrophobicity. Since compounds within the same homologous series exhibit similar properties except volume V (see Abraham descriptors for phenones in Table SI1), all coefficients and descriptors in Eq. (2) can be considered constant, except volume V, which is the only variable, and a conjoint constant ${(r}^{0}$) written according to Eq. (7).

7 $${r}^{0}={10}^{(c+e\mathrm{E}+s\mathrm{S}+a\mathrm{A}+b\mathrm{B})}$$

and the LFER. Abraham Eq. (2) for LEKC mass distribution ratio can be written as in Eq. (8).

8 $${k}_{\mathrm{LEKC}}={r}^{0}{10}^{v\mathrm{V}}$$

The combination of the previous expression with Eq. (1) yielded Eq. (9). Based on the experimental determination of the electrophoretic mobility of the members of any homologous series (various alkylphenones in our case), Eq. (9) serves as a theoretical model for extrapolation to liposome mobility. Non-linear regression of the electrophoretic mobilities of the phenones $({\mu }_{\mathrm{ep}})$ towards their volume (V) provides the constants ${r}^{0}$, *v*, and ${\mu }_{\mathrm{lip}}.$

9 $${\mu }_{\mathrm{ep} }={\mu }_{\mathrm{lip} }\left(1+\frac{1}{{r}^{0}\cdot {10}^{ v\mathrm{V}}}\right)$$

To determine the mobility of the liposome using this fourth method, five alkylphenones were selected: acetophenone, propiophenone, butyrophenone, valerophenone, and hexanophenone. Compounds with longer alkyl chains were not employed, as they are not fully soluble in methanol at 60% (v/v). A single solution containing all five compounds was prepared in methanol and Na_2_HPO_4_ buffer, with the liposome suspension serving as the electrolyte. The fourth method produced clear electropherograms, shown in Fig. 1d, in which methanol and the five alkylphenones are readily distinguishable. The elution order is governed by molecular volume: the larger the volume, the longer the alkylphenone takes to reach the detector. Accordingly, the first peak corresponds to acetophenone, followed sequentially by propiophenone, butyrophenone, valerophenone, and hexanophenone.

**Fig. 2:**
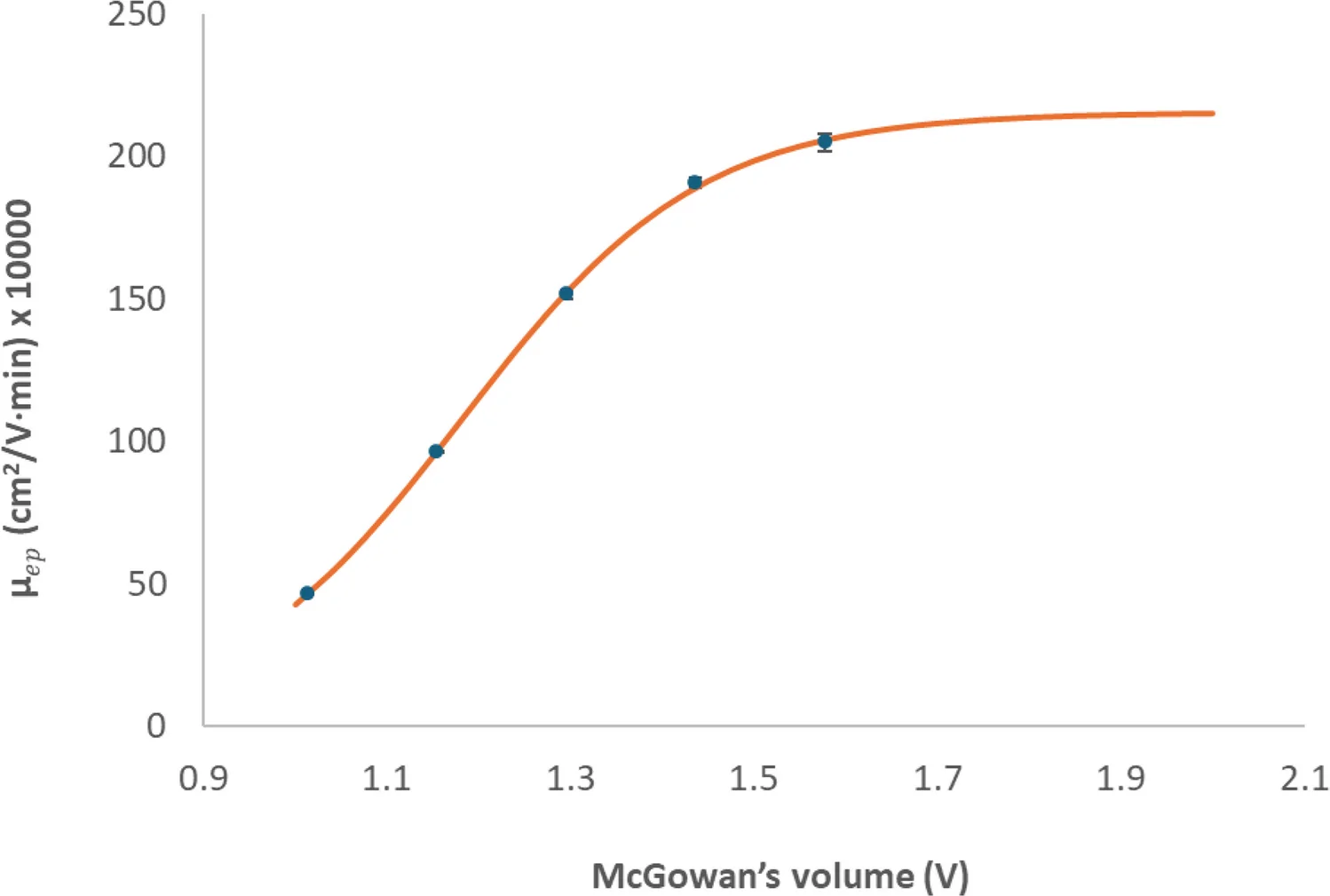
Mobility of homologous alkylphenones according to their McGowan’s volume

Based on the experimental values obtained using the fourth method and applying Eq. (9), the mobility of the liposomes was calculated. Figure 2 shows how the applied model fits the experimental data. Each data point represents the mean of three replicate measurements.

The liposome mobility calculated using the fourth method (215.0 ± 1.0) was very similar to that obtained with the third method, showing a difference of 1.2%. This finding further supports the hypothesis that the supporting electrolyte influences the electrophoretic mobility of the compounds. Nevertheless, the fourth approach was chosen for this study, as it overcomes the solubility and reproducibility limitations observed with the third method.

Prior to performing the measurements required to determine the electrophoretic mobility of various solutes, the mobility of the liposomes was determined daily by Eq. (9) extrapolation. Using the mobilities of both the solutes and the liposomes, the corresponding mass distribution ratios were calculated.

### Abraham characterization of the PC_49_/PA_43_/C_8_ LEKC system

Mass distribution ratios for 37 solutes were calculated to characterize the LEKC system using the Abraham solvation parameter model. Solutes were selected from [30] where several micellar electrokinetic chromatography systems were characterized by means of the same model. Table SI1 lists the compounds along with their Abraham descriptors and log *k* values.

The system coefficients were determined by applying multiple linear regression, regressing solutes’ log* k* values on their descriptors. To detect potential outliers, the standard residuals criterion of rejecting solutes with more than twice the regression standard deviation was applied. According to this criterion, 3-nitroaniline and furan, marked with an asterisk in Table SI1, were identified as outliers and excluded from the regression. The obtained system coefficients, together with their standard error and relevant statistics, are reported in Eq. (10).

10 $$\mathrm{log}k=-1.75\left(0.12\right)+0.22\left(0.16\right)\mathrm{E}-0.61\left(0.12\right)\mathrm{S}+0.30\left(0.09\right)\mathrm{A}-2.73\left(0.23\right)\mathrm{B}+2.91(0.15)\mathrm{V}$$

n = 35; R^2^ = 0.93; SD = 0.15; F = 90.

The coefficient of determination (R^2^) shows that the regression model explains a substantial proportion of the variance in the experimental data. The residual standard deviation (SD) is comparable to that of the experimental measurements, indicating that the model adequately captures their natural variability. The Fisher F-statistic (F) further confirms the overall statistical significance of the model.

Analysis of the individual coefficients revealed that all, except for *e*, are statistically significant at the 95% confidence level according to Student’s t-test. Coefficient* e*, which reflects the medium’s potential to interact with solutes via n-type and π electrons, does not significantly affect retention. This suggests that solutes capable of such interactions behave similarly to those that are not.

Coefficient *s* measures the ability of the system’s phases to establish dipole-dipole and dipole-induced dipole interactions with the solute. It has a negative value, indicating that liposomes are less polar than the aqueous phase, which is consistent with the chemical nature of both phases. Accordingly, solutes with higher dipolarity or polarizability (S) are less likely to be retained.

Coefficient *a* reflects the hydrogen bond basicity of the medium. In this case, it has a positive value, showing that liposomes act more effectively as hydrogen bond acceptors than the aqueous phase. Thus, solutes with hydrogen bond donor groups, i.e., higher hydrogen bond acidity (A), are more strongly retained.

However, the coefficients with the greatest impact on retention are those associated with phases hydrophobicity (*v*) and hydrogen bond acidity (*b*). Coefficient v is positive, indicating that liposomes are more hydrophobic than the aqueous phase, so retention increases with solute McGowan volume (V). In contrast, coefficient* b* is negative, reflecting the greater ability of the aqueous phase to act as a hydrogen bond donor. Consequently, solutes with hydrogen bond acceptor groups, i.e., higher hydrogen bond basicity (B), are less strongly retained within liposomes.

### Comparison of the PC_49_/PA_43_/C_8_ LEKC system to biological blood/organ distributions

The feasibility of the surrogation of partition in biological systems was tested by comparison of the system constants of the characterized PC_49_/PA_43_/C_8_ system to several in vivo and in vitro biological blood-tissue distributions by passive partition previously characterized by the same Abraham model [31]. Table 1 summarizes the normalized coefficients and the calculated distances between LEKC and biological systems. The compared biological systems include in vivo partitions of drugs from blood to eight different rat’s tissues and seven in vitro blood-tissue partitions, which were supposed to surrogate the corresponding in vivo partitions. The partition data for these in vitro partitions was calculated from saline-blood (usually plasma) and saline-tissue partitions.

**Table 1:** Constant term, module and normalized system constants of the LEKC and the compared blood/organ partition biological systems with the *d* distance to LEKC system

System	*c*	l	${e}_{u}$	${s}_{u}$	${a}_{u}$	${b}_{u}$	${v}_{u}$	*d*
PC_49_/PA_43_/C_8_	−1.75	4.05	0.06	−0.15	0.07	−0.67	0.72	0.00
Blood-brain in vivo	0.55	1.30	0.17	−0.46	−0.49	−0.52	0.49	0.71
Blood-muscle in vivo	0.08	0.28	−0.21	0.04	−0.89	0.10	0.39	1.32
Blood-liver in vivo	0.29	0.59	0.00	−0.50	−0.57	0.31	0.57	1.23
Blood-lung in vivo	0.27	1.15	0.00	−0.46	−0.63	0.00	0.63	1.02
Blood-kidney in vivo	0.49	0.74	−0.09	−0.58	−0.50	0.31	0.56	1.23
Blood-heart in vivo	0.13	0.76	−0.05	−0.52	−0.50	0.01	0.69	0.97
Blood-skin in vivo	−0.11	1.02	−0.11	0.03	0.00	−0.66	0.74	0.26
Blood-fat in vivo	0.08	2.18	0.11	−0.10	−0.41	−0.70	0.57	0.51
Blood-brain in vitro	−0.06	1.03	0.02	−0.54	−0.31	−0.32	0.71	0.65
Blood-muscle in vitro	−0.19	0.99	−0.21	−0.60	−0.08	−0.17	0.75	0.74
Blood-liver in vitro	−0.10	0.91	0.00	−0.40	−0.39	−0.20	0.80	0.71
Blood-lung in vitro	−0.14	0.49	0.00	0.00	0.00	−0.78	0.63	0.23
Blood-kidney in vitro	−0.16	1.31	0.15	−0.35	−0.70	0.18	0.57	1.18
Blood-heart in vitro	0.05	0.98	0.04	−0.05	0.08	−0.23	0.97	0.52
Blood-fat in vitro	0.47	3.16	0.01	0.00	−0.50	−0.71	0.49	0.64

Based on the relative arbitrary criterion that a distance about 0.25 or less indicates an acceptable or good surrogation of the biological system by the physicochemical one [12] it turns out that the LEKC system can be an acceptable surrogation system for the in vivo blood-skin partition (*d* = 0.26). The in vitro blood-lung partition shows even a closer similarity to the PC_49_/PA_43_/C_8_ system (*d* = 0.23). However, the corresponding in vivo blood-lung partition presents a large distance to the LEKC system (*d* = 1.01), supporting Abraham and co-workers’ findings that some apparently reasonable in vitro systems may not be good surrogates of their corresponding in vivo systems [31].

**Fig. 3:**
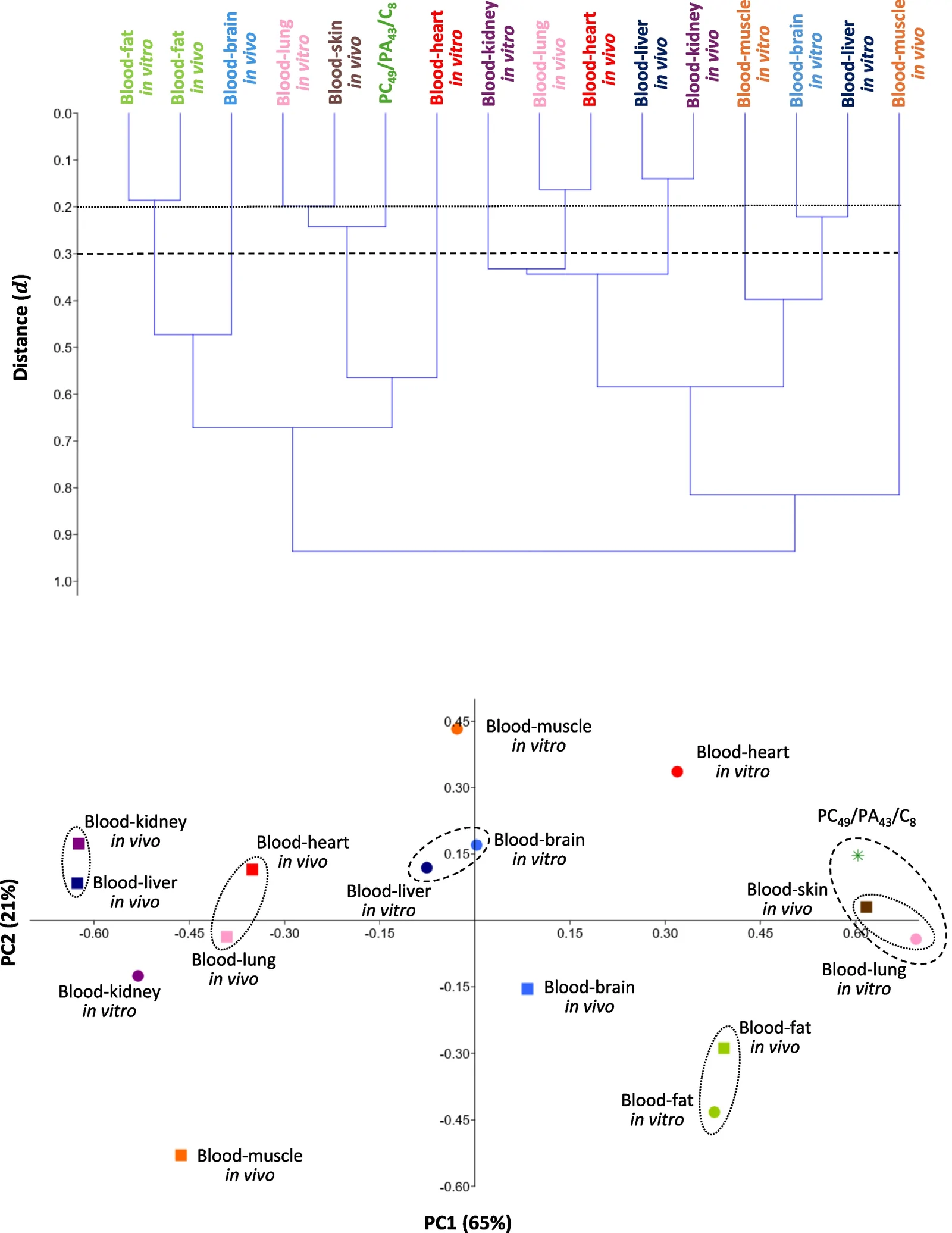
Dendrogram and main PCA plot of the LEKC and biological compared systems

Dendrograms and a PCAs have been proposed to clarify the similarities and discrepancies between in vivo and surrogate in vitro systems [12]. They are presented in Fig. 3 for the compared LEKC, in vitro, and in vivo systems.

In the PCA, the first principal component (PC1) explains 65% of the variance, while the second principal component (PC2) accounts for 21%. The coefficient with the highest weight in PC1 corresponds to hydrogen bond acidity (*b*), whereas in PC2 it corresponds to hydrogen bond basicity (*a*) (see Table SI3 in the Supplementary Information). Accordingly, the distribution of the systems along PC1 is primarily governed by hydrogen bond acidity, while PC2 mainly reflects differences in hydrogen bond basicity. These results indicate that hydrogen bonding interactions are the dominant factors controlling system similarity and therefore play a key role in the assessment of surrogation across LEKC, in vitro, and in vivo systems. On this basis, modification of system composition may allow adjustment of these parameters to better reproduce the target biological environment.

Two clustering limits have been set in both the dendrogram and the PCA: clusters with *d* < 0.20 with systems expected to be well correlated, and clusters with *d* < 0.30 of systems with an expected acceptable correlation.

It is noteworthy that the unique in vitro system expected to be a good surrogate of the corresponding in vivo system is blood-fat partition with *d* = 0.19. All other in vitro partitions are not similar enough to the same in vivo partitions to be good surrogates (*d* distances between 0.36 for blood-lung and 1.34 for blood-kidney).

It is also noticeable that some in vivo and in vitro partitions are very similar. Thus, in vivo blood-liver is very close to in vivo blood-kidney (*d* = 0.14) and in vivo blood-lung to in vivo blood-heart (*d* = 0.16). In vitro blood-brain is quite similar to in vitro blood-liver (*d* = 0.22).

Regarding the studied PC_49_/PA_43_/C_8_ system, it is quite similar (*d* ≈ 0.25) to the cluster formed by in vitro blood-lung and in vivo blood-skin (*d* = 0.20), confirming it can be a good surrogate of these systems.

## Conclusions

Liposome electrokinetic chromatography can be a good tool to mimic the composition of cell membranes and thus surrogate biopartitions with the advantages of low sample and reagent consumption, high separation efficiency, and short analysis time. However, it requires hydrophobic markers to determine the liposome mobility, which are hardly soluble in the medium. The proposed new method based on the extrapolation of the mobility of soluble homologous series members can be an attractive alternative to avoid this problem. In addition, the relatively narrow elution window of LEKC can cause highly hydrophilic and hydrophobic compounds to co-elute with the electroosmotic flow and liposome markers, respectively. Nevertheless, compounds with intermediate hydrophilic-hydrophobic character can be reliably analyzed.

Characterization of a LEKC system with a cell membrane representative liposome composed of phosphatidylcholine (49%), palmitic acid (43%), and cholesterol (8%) by the general LFER Abraham solvation parameter model reveals that the main interactions that determine distribution between the liposome and the carrier aqueous phases are the creation of solute cavities in the phases, which favors partition to the liposome, and the hydrogen bonding from phases to the solute, which favors partition to the aqueous phase. In a minor degree hydrogen bonding and dipolarity/polarizability interactions from solute to phases are also relevant. Unexpectedly, hydrogen bonding from solute to liposome is found to be stronger than hydrogen bonding to water.

Comparison of the solute-liposome interactions with those of several in vivo and in vitro blood-organ partitions, characterized by the same Abraham model, indicates that the liposome can be a good surrogate to determine blood-skin partitions. Moreover, a cluster analysis of the interactions in the LEKC partition system and in vivo and in vitro blood-organ distributions shows that many in vitro distributions do not surrogate well their corresponding in vivo distributions. Therefore, we propose the characterization of the in vivo and expected in vitro surrogation systems by the same model (e.g., Abraham model) and analysis of their similarity to test the feasibility of surrogation.

## Supplementary Information

Below is the link to the electronic supplementary material.

Supplementary file1 (DOCX 35.6 KB)

## Data Availability

Data will be made available on request.

## References

[1] Tsopelas F, Stergiopoulos C, Danias P, Tsantili-Kakoulidou A. Biomimetic separations in chemistry and life sciences. Mikrochim Acta. 2025;192: 133. 10.1007/s00604-025-06980-x. 39904888 PMC11794418

[2] Valko KL. Biomimetic chromatography-a novel application of the chromatographic principles. Anal Sci Adv. 2022;3:146–53. 10.1002/ansa.202200004. 38715641 PMC10989578

[3] Valko KL. Application of biomimetic HPLC to estimate *in vivo* behavior of early drug discovery compounds. Future Drug Discov. 2019;1: FDD11. 10.4155/fdd-2019-0004.

[4] Roy K. Cheminformatics, QSAR and machine learning applications for novel drug development. Academic Press. 2023. 10.1016/C2022-0-00080-5.

[5] Stergiopoulos C, Tsopelas F, Valko K. Prediction of hERG inhibition of drug discovery compounds using biomimetic HPLC measurements. ADMET DMPK. 2021;9:191–207. 10.5599/admet.995. 35300361 PMC8920097

[6] Lishchuk V, Wiedmer SK. Recent trends in capillary electrokinetic chromatography with liposomes, lipid aggregates, and lipid emulsions. J Chromatogr Open. 2024;6: 100163. 10.1016/j.jcoa.2024.100163.

[7] Wang Y, Sun J, Liu H, et al. Prediction of human drug absorption using liposome electrokinetic chromatography. Chromatographia. 2007;65:173–7. 10.1365/s10337-006-0140-3.

[8] Zhang K, Fahr A, Abraham MH, et al. Comparison of lipid membrane-water partitioning with various organic solvent-water partitions of neutral species and ionic species: uniqueness of cerasome as a model for the stratum corneum in partition processes. Int J Pharm. 2015;494:1–8. 10.1016/j.ijpharm.2015.08.010. 26256152

[9] Amézqueta S, Fernández-Pumarega A, Farré S, et al. Lecithin liposomes and microemulsions as new chromatographic phases. J Chromatogr A. 2020;1611: 460596. 10.1016/j.chroma.2019.460596. 31610920

[10] Orzel D, Ravald H, Dillon A, et al. Immobilised artificial membrane liquid chromatography vs liposome electrokinetic capillary chromatography: suitability in drug/bio membrane partitioning studies and effectiveness in the assessment of the passage of drugs through the respiratory mucosa. J Chromatogr A. 2024;1734: 465286. 10.1016/j.chroma.2024.465286. 39191185

[11] Štěpánová S, Kašička V. Determination of physicochemical parameters of (bio)molecules and (bio)particles by capillary electromigration methods. J Sep Sci. 2024;47: e2400174. 10.1002/jssc.202400174. 38867483

[12] Fuguet E, Rosés M. Tutorial on modelling chromatographic surrogation of biological processes. J Chromatogr Open. 2024;6: 100189. 10.1016/j.jcoa.2024.100189.

[13] Dobrzyńska I, Szachowicz-Petelska B, Sułkowski S, Figaszewski Z. Changes in electric charge and phospholipids composition in human colorectal cancer cells. Mol Cell Biochem. 2005;276:113–9. 10.1007/s11010-005-3557-3. 16132692

[14] Hancock SE, Friedrich MG, Mitchell TW, et al. The phospholipid composition of the human entorhinal cortex remains relatively stable over 80 years of adult aging. GeroScience. 2017;39:73–82. 10.1007/s11357-017-9961-2. 28299641 PMC5352591

[15] Fu J, Ding X, Stowell CET, et al. Slow degrading poly(glycerol sebacate) derivatives improve vascular graft remodeling in a rat carotid artery interposition model. Biomaterials. 2020;257: 120251. 10.1016/j.biomaterials.2020.120251. 32738658 PMC8422746

[16] Cui Z-K, Bastiat G, Jin C, et al. Influence of the nature of the sterol on the behavior of palmitic acid/sterol mixtures and their derived liposomes. Biochim Biophys Acta. 2010;1798:1144–52. 10.1016/j.bbamem.2010.02.008. 20153720

[17] Riekkola M-L, Jönsson JÅ, Smith RM. Terminology for analytical capillary electromigration techniques (IUPAC recommendations 2003). Pure Appl Chem. 2004;76:443–51. 10.1351/pac200476020443.

[18] Abraham MH. Scales of solute hydrogen-bonding: their construction and application to physicochemical and biochemical processes. Chem Soc Rev. 1993;22: 73. 10.1039/cs9932200073.

[19] Poole CF. Solvation parameter model: tutorial on its application to separation systems for neutral compounds. J Chromatogr A. 2021;1645: 462108. 10.1016/j.chroma.2021.462108. 33857674

[20] UFZ - LSER database. https://www.ufz.de/lserd. Accessed 27 June 2025.

[21] ACD/Percepta. ACD/Labs 2014 Release. Advanced Chemistry Development, Inc. Toronto (Canada).(n.d.). www.acdlabs.

[22] Liu X, Acree WE Jr., Abraham MH. Descriptors for some compounds with pharmacological activity; calculation of properties. Int J Pharm. 2022;617: 121597. 10.1016/j.ijpharm.2022.121597. 35181462

[23] Acree B. Toxicity and drug testing. INTECH. 2012. 10.5772/1976.

[24] Beltrán JL, Pignatello JJ, Teixidó M. ISOT_calc: a versatile tool for parameter estimation in sorption isotherms. Comput Geosci. 2016;94:11–7. 10.1016/j.cageo.2016.04.008.

[25] Hammer Ø, Harper D, Ryan P. Past: paleontological statistical software package for education and data analysis. Palaeontol Electron. 2001;4:1–9.

[26] Perera D, Lishchuk V, Moghaddam AH, et al. Differences in the distribution of steroids in sterol-containing liposomes: a study by liposome electrokinetic chromatography. J Chromatogr A. 2025;1743: 465688. 10.1016/j.chroma.2025.465688. 39837185

[27] Fernández-Pumarega A, Amézqueta S, Fuguet E, Rosés M. Determination of the retention factor of ionizable compounds in microemulsion electrokinetic chromatography. Anal Chim Acta. 2019;1078:221–30. 10.1016/j.aca.2019.06.007. 31358222

[28] Subirats X, Justicia A, Rosés M. Chasing the elusive hold-up time from an LFER approach. J Chromatogr A. 2018;1571:176–84. 10.1016/j.chroma.2018.08.017. 30150116

[29] Redón L, Subirats X, Rosés M. Evaluation of hold-up volume determination methods and markers in Hydrophilic Interaction Liquid Chromatography. Molecules. 2023;28: 1372. 10.3390/molecules28031372. 36771038 PMC9920175

[30] Fuguet E, Ràfols C, Bosch E, et al. Solute-solvent interactions in micellar electrokinetic chromatography. III. Characterization of the selectivity of micellar electrokinetic chromatography systems. J Chromatogr A. 2002;942:237–48. 10.1016/s0021-9673(01)01383-8. 11822389

[31] Abraham MH, Gola JMR, Ibrahim A, et al. The prediction of blood-tissue partitions, water-skin partitions and skin permeation for agrochemicals: Prediction of blood-tissue partitions, water-skin partitions and skin permeation. Pest Manag Sci. 2014;70:1130–7. 10.1002/ps.3658. 24085512

