# Supplementary material for: Characterization of a liposome electrokinetic capillary chromatography system for surrogation of cell membrane partitions
Source: Anal Bioanal Chem. 2026 Mar 3;418(18):5895–905. doi: 10.1007/s00216-026-06365-w (PMC13541782; doi:10.1007/s00216-026-06365-w)
Supplement: Supplementary file 1 [file 216_2026_6365_MOESM1_ESM.docx]

**Supplementary Information**

**Characterization of a liposome electrokinetic capillary chromatography system for surrogation of cell membrane partitions**

Paula Vidal^1^, Elisabet Fuguet^1,2^, Martí Rosés^1^*

^1^Department of Analytical Chemistry and Institute of Biomedicine of the University of Barcelona (IBUB), Universitat de Barcelona, Martí i Franquès 1-11, E-08028 Barcelona, Spain

^2^Serra Húnter Fellow, Generalitat de Catalunya, Spain

*Corresponding author Martí Rosés: marti.roses@ub.edu

Table SI1	Abraham descriptors and mass distribution ratio of the characterization solutes

| **Solute** | ***E*** | ***S*** | ***A*** | ***B*** | ***V*** | **log *k*** |
| --- | --- | --- | --- | --- | --- | --- |
| Acetophenone | 0.82 | 1.01 | 0.00 | 0.48 | 1.0139 | -0.56 |
| Propiophenone | 0.80 | 0.95 | 0.00 | 0.51 | 1.1548 | -0.09 |
| Butyrophenone | 0.80 | 0.95 | 0.00 | 0.51 | 1.2957 | 0.38 |
| Valerophenone | 0.80 | 0.95 | 0.00 | 0.50 | 1.4366 | 0.89 |
| Hexanophenone | 0.78 | 1.15 | 0.00 | 0.44 | 1.5775 | 1.08 |
| Benzamide | 0.99 | 1.50 | 0.49 | 0.67 | 0.9728 | -1.15 |
| Pyrrole | 0.61 | 0.91 | 0.22 | 0.25 | 0.5774 | -1.10 |
| Hydroquinone | 1.06 | 1.27 | 1.06 | 0.57 | 0.8338 | -1.03 |
| 3-Nitroaniline* | 1.20 | 1.71 | 0.40 | 0.35 | 0.9904 | -1.00 |
| Aniline | 0.96 | 0.96 | 0.26 | 0.41 | 0.8162 | -0.93 |
| Benzaldehyde | 0.82 | 1.00 | 0.00 | 0.39 | 0.8730 | -0.79 |
| Benzonitrile | 0.74 | 1.11 | 0.00 | 0.33 | 0.8711 | -0.72 |
| Phenol | 0.81 | 0.89 | 0.60 | 0.30 | 0.7751 | -0.70 |
| Resorcinol | 0.98 | 1.11 | 1.09 | 0.52 | 0.8338 | -0.69 |
| o-Toluidine | 0.97 | 0.92 | 0.23 | 0.45 | 0.9571 | -0.67 |
| Quinoline | 1.27 | 0.97 | 0.00 | 0.54 | 1.0443 | -0.53 |
| 4-Nitroaniline | 1.22 | 1.93 | 0.46 | 0.35 | 0.9904 | -0.52 |
| Acetanilide | 0.90 | 1.39 | 0.48 | 0.67 | 1.1137 | -0.50 |
| Cortisone | 1.96 | 3.50 | 0.36 | 1.87 | 2.7546 | -0.45 |
| Nitrobenzene | 0.87 | 1.11 | 0.00 | 0.28 | 0.8906 | -0.40 |
| Monuron | 1.14 | 1.50 | 0.47 | 0.78 | 1.4768 | -0.35 |
| 3-Methylphenol | 0.82 | 0.88 | 0.57 | 0.34 | 0.9160 | -0.35 |
| Hydrocortisone | 2.03 | 3.49 | 0.71 | 1.90 | 2.7976 | -0.31 |
| 2-Nitroanisole | 0.97 | 1.34 | 0.00 | 0.45 | 1.0902 | -0.30 |
| Anisole | 0.71 | 0.75 | 0.00 | 0.29 | 0.9160 | -0.26 |
| 4-Chloroaniline | 1.06 | 1.13 | 0.30 | 0.31 | 0.9386 | -0.23 |
| 2-Nitroaniline | 1.18 | 1.37 | 0.30 | 0.36 | 0.9904 | -0.18 |
| 4-Chloroacetanilide | 0.98 | 1.47 | 0.64 | 0.51 | 1.2361 | -0.12 |
| Methyl benzoate | 0.73 | 0.85 | 0.00 | 0.46 | 1.0726 | -0.09 |
| 2,4-Dimethylphenol | 0.84 | 0.80 | 0.53 | 0.39 | 1.0569 | -0.04 |
| 2,3-Dimethylphenol | 0.85 | 0.85 | 0.52 | 0.36 | 1.0569 | -0.02 |
| 4-Chlorophenol | 0.92 | 1.08 | 0.67 | 0.20 | 0.8975 | 0.00 |
| Benzophenone | 1.45 | 1.50 | 0.00 | 0.50 | 1.4080 | 0.41 |
| Furan* | 0.37 | 0.51 | 0.00 | 0.13 | 0.5363 | 0.44 |
| 2-Naphthol | 1.52 | 1.08 | 0.61 | 0.40 | 1.1441 | 0.54 |
| Geraniol | 0.51 | 0.63 | 0.39 | 0.66 | 1.4903 | 0.65 |
| Thymol | 0.82 | 0.79 | 0.52 | 0.44 | 1.3387 | 0.99 |

* Outlier solutes.

**Liposome stability study**

To assess the stability of the liposome system, a study was performed to evaluate changes in liposome size over time and their impact on electrophoretic mobility. Using LEKC and Eq. (6), the electrophoretic mobility of acetophenone, propiophenone, and butyrophenone was measured over two consecutive days, with methanol used as the electroosmotic flow marker.

Liposome size was determined prior to each analysis. Three measurements were carried out: on the day of liposome preparation, and on the following day, before and after re-extrusion of the suspension (five passes through 100 nm and five passes through 50 nm filters). Table SI2 summarizes the liposome sizes and the corresponding electrophoretic mobilities.

**Table SI2.** Effect of liposome size on electrophoretic mobilities

|  |  | $\mu_{\mathrm{ep}}(\mathrm{cm}^{2}/V\cdot min) x 10000$ | | |
| --- | --- | --- | --- | --- |
| Condition | Liposome size (nm) | Acetophenone | Propiophenone | Butyrophenone |
| Initial | 74.1 ± 0.9 | 50.4 ± 0.6 | 96 ± 2 | 144 ± 3 |
| Before extrusion | 201 ± 4 | 36.4 ± 0.1 | 77.0 ± 0.1 | 122.5 ± 0.2 |
| After extrusion | 66.9 ± 0.5 | 49.4 ± 0.1 | 103 ± 2 | 153.5 ± 0.5 |

As shown, after overnight storage at room temperature, liposome size increased from 74.1 nm to 201 nm, while re-extrusion restored the size to 66.9 nm. Although the electrophoretic mobilities for liposomes of 74.1 nm and 66.9 nm were generally similar, minor differences were observed. In contrast, the mobilities obtained with larger liposomes (201 nm) were significantly lower. Consequently, a single liposome mobility value ($\mu_{\mathrm{lip}}$) was not assumed and was instead determined daily to ensure accurate mass distribution ratios (*k*).

Table SI3	Loadings of the different principal components

|  | PC1 | PC2 | PC3 | PC4 | PC5 |
| --- | --- | --- | --- | --- | --- |
| $e_{u}$ | 0.04 | -0.06 | -0.29 | 0.90 | 0.31 |
| $s_{u}$ | 0.34 | -0.50 | 0.78 | 0.19 | 0.03 |
| $a_{u}$ | 0.50 | 0.70 | 0.22 | -0.07 | 0.46 |
| $b_{u}$ | -0.79 | 0.28 | 0.48 | 0.14 | 0.22 |
| $v_{u}$ | 0.10 | 0.44 | 0.18 | 0.36 | -0.80 |
